# Alinéa suicide postvention program: a codesigned early proactive intervention for survivors

**DOI:** 10.3389/fpsyg.2024.1436680

**Published:** 2024-12-09

**Authors:** Mélanie Coquelin, Céline Kopp-Bigault, Canelle Barinoil, Sofian Berrouiguet, Cinzia Guarnaccia

**Affiliations:** ^1^Lp3c (Laboratoire de Psychologie: Cognition, Comportement, Communication), Université Rennes 2, Rennes, France; ^2^Alinéa, Fondation Bon Sauveur, Bégard, France; ^3^Centre de recherches en Epidémiologie et Santé des Populations, Equipe INSERM MOODS U1018, Le Kremlin Bicêtre, Université de Paris Saclay, Paris, France; ^4^LaTIM, INSERM, UMR 1101, Université de Brest, Brest, France

**Keywords:** Alinéa, suicide, active postvention, bereavement, first responders, protocol

## Abstract

**Background:**

Bereavement following suicide is a risk factor for major depression, post-traumatic stress disorder, suicidal behavior, the emergence of bipolar disorders and prolonged mourning. The scientific literature agrees on the need to deploy support services tailored to the specificities of post-suicide bereavement. Alinéa responds to these specificities with an early and proactive individual postvention program.

**Method:**

This article presents the process of co-designing Alinéa by involving participants who have been exposed to suicide either through the loss of a loved one or in the course of their work. This co-design led to the creation of a collaborative system for early, proactive professional intervention. The Alinéa program, design intervention objectives and protocol, are presented in this article.

**Discussion:**

The Alinéa system is committed to research and is developing different types of research: evaluative research, fundamental research and action research.

## Introduction

### The specific needs of people bereaved by suicide

#### A specific form of bereavement

People bereaved by suicide, often referred to as “suicide survivors,” are most frequently family members and close friends of the victim (Cerel et al., 2014). Bereavement following suicide differs from other types of bereavement in terms of its intensity and complexity, with suicide survivors experiencing higher levels of suffering, anger, and guilt, compounded by feelings of shame, social stigma, and physical and psychological complications (Peters et al., 2016; Spillane et al., 2018). Consequently, bereavement by suicide is a major risk factor for depression, posttraumatic stress, suicidal behaviors, and prolonged grief, and is capable of triggering bipolar disorders (Groot et al., 2006; Harwood et al., 2002; Jordan, 2017; Young et al., 2012). According to one estimate, every death by suicide has a lasting impact on 14 people (Crosby and Sacks, 2002).

#### Early and proactive intervention

People bereaved by suicide need specific types of support (Andriessen et al., 2017), which can be provided in two ways:

*Early intervention* during the first few days or weeks after the death. Early interventions aim to preempt and reduce personal and interpersonal complications after suicide. When suffering the stress and psychological distress caused by a loved one’s suicide, the bereaved need early support tailored to their individual and family experiences. This support must include information about the help available, ways of talking to children about the death, and administrative procedures (Harwood et al., 2002; McGill et al., 2023). Recommendations for how long support should continue vary from 6 to 24 months after the death to as long as is necessary for the bereaved person’s general state to stabilize (Dyregrov, 2011; McGill et al., 2023).

*Proactive intervention*, also known as active postvention, differs from passive postvention in that it involves support services initiating contact with the bereaved (Campbell et al., 2004). Because individual and interpersonal complications (Andriessen and Krysinska, 2012; Dyregrov, 2011) can make it difficult for suicide survivors to ask for help (Feigelman et al., 2009; Peters et al., 2016; Entilli et al., 2021), they often do not seek support until 4 or 5 years after the death (Campbell et al., 2004). By taking the first step in offering support, active postvention can meet the needs of bereaved people who feel unable to ask for help and thereby prevent or reduce their psychological and social isolation.

Given suicide survivors’ reluctance to ask for help, early intervention must be proactive. The main challenge of early and proactive intervention is regulatory access to information: death by suicide and contact with relatives. Studies have shown the influence of several factors, among which we can cite the role of social and cultural factors (Raj et al., 2024). To achieve this, support services must work closely with law enforcement (McGeechan et al., 2018; Duval et al., 2022).

Early proactive support can take various forms:

*Intervention at the place of death* aims to reduce the effects of stress by providing immediate support. This is the goal of active postvention models, the best known of which is Loving Outreach Survivors of Suicide (LOSS), created in Baton Rouge in the United States (Campbell et al., 2004).

*Home visits*, such as those provided by Australia’s Living Beyond Aboriginal Suicide service (Goodwin-Smith et al., 2013), enable support workers to offer practical guidance immediately after a death.

*Telephone calls* are generally the first step in setting up a telephone monitoring program lasting several months. Examples of this type of intervention include the United States’ Tragedy Assistance Program for Survivors, aimed at members of the armed services (Ruocco et al., 2022), and Italy’s SOPRoxi program (Scocco et al., 2017). Telephone monitoring programs are also effective in preventing suicide, as they enable support services to stay in contact with people who have tried to take their own lives (Duhem et al., 2018).

*Letters* enable support services to offer condolences in the days following the death and to inform the bereaved about available support. The Netherland’s Safecare program, designed for people bereaved by a suicide on the rail network, uses this approach (Van der Veer, 2017).

#### Identifying and evaluating existing services

McDonnell et al.’s 2017–2018 survey of the experiences of 7,158 British people bereaved by suicide confirmed suicide’s impacts on survivors and the need for immediate and proactive professional support to reduce subsequent health problems, negative social consequences, and suicidal behaviors (McDonnell et al., 2022). Following, respectively, an audit of suicide cases in Montreal, Canada, and a qualitative study of the experiences of people bereaved by suicide in Queensland, Australia, both Ligier et al. (2020) and Ross et al. (2021) highlighted the importance of developing proactive services with the flexibility to respond to survivors’ differing needs at different points in the bereavement trajectory. However, such services are rare, and they have rarely been evaluated. Indeed, McGill et al. (2023) found only 11 studies measuring the effectiveness of individual postvention programs during the last 35 years. Programs incorporating the research-based recommendation that support should be proactive, early, flexible, and collaborative with first responders are also rare (Coquelin et al. 2015). Evaluating programs is essential to identify which actions are effective and their modes of impact (McGill et al., 2023; Jordan, 2017). In line with this need, this article aims to describe the design and the implementation of a postvention program (Alinéa).

## Context and method

### Alinéa

Alinéa is an early proactive suicide postvention service provided by professional counselors (psychologists and nurses), in collaboration with law enforcement (first responders). Its protocol was codesigned, incorporating input from several fields, and then rolled out for testing and evaluation in 2021. The following sections describe Alinéa’s design process, objectives, and protocol.

### Codesign

#### The codesign principle

User experiences are an effective factual and contextual basis for developing new healthcare strategies (Cluley et al., 2022). Experience-based design (Robert and Bate, 2023), for example, involves working with users to develop innovations that meet users’ needs and objectives and to ensure they embrace the results (Holland-Hart et al., 2019). The user-codesigner’s role evolves throughout the codesign process and includes questioning, informing, listening, providing feedback, and giving advice (Robert and Bate, 2023). Krysinska et al. (2023) advocated using coproduction in suicide research by involving people with lived experience of suicide. The Alinéa codesign process heeded this recommendation by including people bereaved by suicide and professionals frequently involved in cases of suicide.

#### The Alinéa codesign process

**Figure 1:**
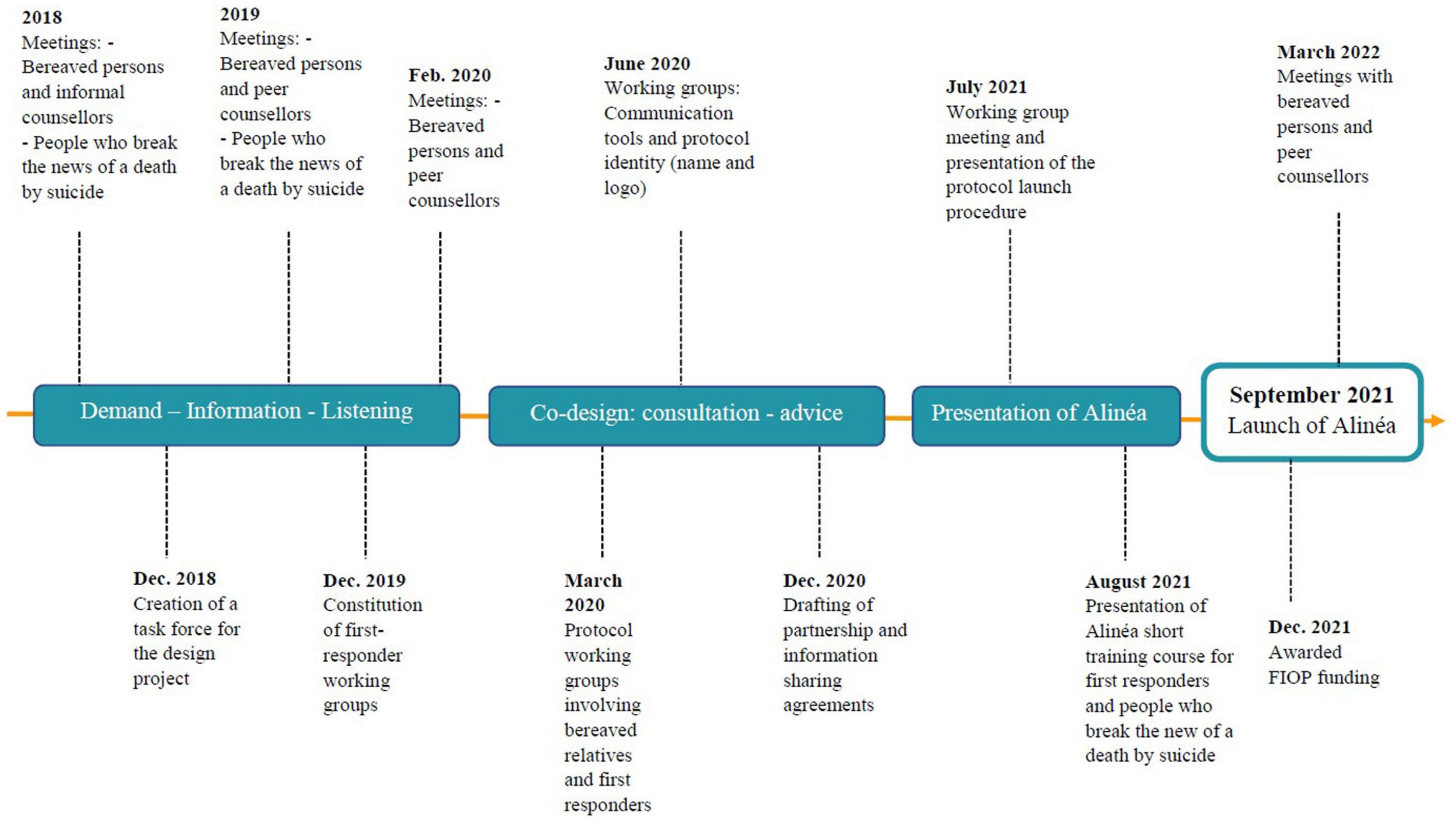
Alinéa co-design timeline.

Alinéa was codesigned (see Figure 1) in the Côtes d’Armor *département* of France, which has the highest suicide rate of all France’s *départements* (24.1 suicides for 100,000 inhabitants, compared with a national average of 12.7 suicides for 100,000 inhabitants) (Observatoire Régional de la Santé en Bretagne, 2022). In the light of this fact, in 2018 suicide-prevention professionals in Côtes-d’Armor began holding discussion groups with people bereaved by suicide. Ten large-group sessions, between 60 and 95 participants, were led by professionals accompanied by a suicide survivor. Participants attended a session following a press release inviting them to “participate in discussions on bereavement after suicide.” Each session was the subject of a thematic restitution by the group facilitators. The issues raised most frequently by the almost 700 suicide survivors who shared their experiences and needs were the feeling of isolation, the lack of specific professional support services, the difficulty in obtaining specialist help, and the need to have a space in which to continue sharing their experiences. The discussion groups also included representatives of support organizations and volunteer-run helplines, who described these services’ limitations and the lack of dedicated professional support. At the end of the group sessions, individual interviews were used to complete the identified themes. This set of information was treated as a basis for discussion.

The next stage involved setting up three working groups comprising people bereaved by suicide, peer caregivers, and first responders involved in cases of suicide (people who break the news of the death, police, paramedics). 25 participants were recruited following a call for volunteers among the partner institutions of the Fondation Bon Sauveur. The objective presented was “to think together about a system for monitoring relatives bereaved by suicide.” Their discussions allowed us to establish timelines of their experiences of suicide’s emotional impacts and practical implications.

For example, as one suicide survivor said: “*My husband and I were sitting on the couch, we could not sleep, we could not eat, someone had to come to you, where we were. As soon as possible*.” A police officer adds: *“We sometimes arrive in a house where there is still life, and we drop a bomb: “your loved one committed suicide.” Suggesting something allows us to go from being a “crisis trigger” to a “bearer of hope.” We all need a way out.”*

We used these findings to determine core features for the postvention service: It had to focus solely on suicide-related grief, so its purpose was clear; it had to be proactive and provide early support to all suicide survivors; and it had to include support for people who break the news of a suicide. We then looked for services in France that had these core features, but we were unable to find any described in the scientific literature. Examination of Canadian and American suicide postvention programs showed that they could not be transposed to France because of procedural differences between North America and France: In North America, suspected cases of suicide are always referred to the coroner’s office before the official cause of death is announced, but this is not the case in France. Consequently, it was decided that the Alinéa model would be tested and evaluated in a specific region, but that it would have to be transferable to other parts of France.

The co-design of Alinéa extended over 2 years. Once the Alinéa protocol had been created, communication materials (leaflet, logo, illustration, name) were designed in conjunction with the suicide survivors in the working groups, first responders, and partners involved in publicizing the service within the region. Alinéa was launched in September 2021.

## The Alinéa design

### The objectives

Alinéa’s overall objective is to reduce the individual and family complications that frequently occur following a suicide. To this end, Alinéa provides support for people bereaved by suicide and training for first responders to deaths by suicide.

### For people bereaved by suicide

Alinéa includes two distinct types of intervention: *monitoring* and *support*.

*Monitoring* is the early, proactive part of the program. It begins with a law enforcement following an alert protocol that includes informing the bereaved that specialist support is available. The alert protocol also requires law enforcement to provide Alinéa with contact details for the bereaved, so an Alinéa counselor (nurse or psychologist) can contact them by telephone within 15 days of the death.

*Support* is available to anyone who asks Alinéa for help at any time following a loved one’s death by suicide. Alinéa also offers support, in the form of individual or family consultations, to people within the monitoring program who experience complications. People eligible for support from Alinéa include members of the suicide victim’s family (children, adolescents, and adults) and the victim’s friends.

### For first responders

As part of the collaboration with law enforcement, the Alinéa team meets each police unit once a year (2 h). Each visit includes awareness-raising on how to announce a death by suicide and presentation of the Alinéa protocol. Reflex sheets have also been created by Alinéa, in collaboration with law enforcement, to guide the first responder in this process.

Law enforcement can also benefit from a 3-h training course on bereavement and the announcement of a sudden death, in the company of elected officials. Elected officials who may accompany police officers to cases of suicide are also offered training in how to break the news of a death by suicide. Training for funeral services is also being developed.

All first responders can contact the Alinéa team to discuss after suicide interventions. Alinéa is presented as a monitoring and support system for relatives bereaved by suicide, and as a support for first responders.

## The Alinéa process

### Ways of accessing the program

Early proactive intervention requires close collaboration with the law enforcement that systematically investigates to determine the cause of death, which allows for systematic linkage between relatives and Alinéa. When suicide is determined as the cause of death, the police officer who informs the family provides them with a document containing information about Alinéa and the support available. This document is also a tool police can draw on to break the news or to give support. The police officer explains to the bereaved that an Alinéa counselor will contact them in the coming days unless they would prefer not to be contacted. If the bereaved agree to be contacted, the officer completes an “alert sheet” containing the names and telephone numbers of the bereaved people present, the manner of death, and how the body was found. Alinéa sets in motion the monitoring protocol as soon as it receives this alert sheet. To ensure police follow the protocol in every case, and to avoid oversights, the local gendarmerie’s family protection service (Maison de Protection des Familles) oversees the process by exchanging information with Alinéa every 2 weeks.

### The Alinéa protocol

**Figure 2:**
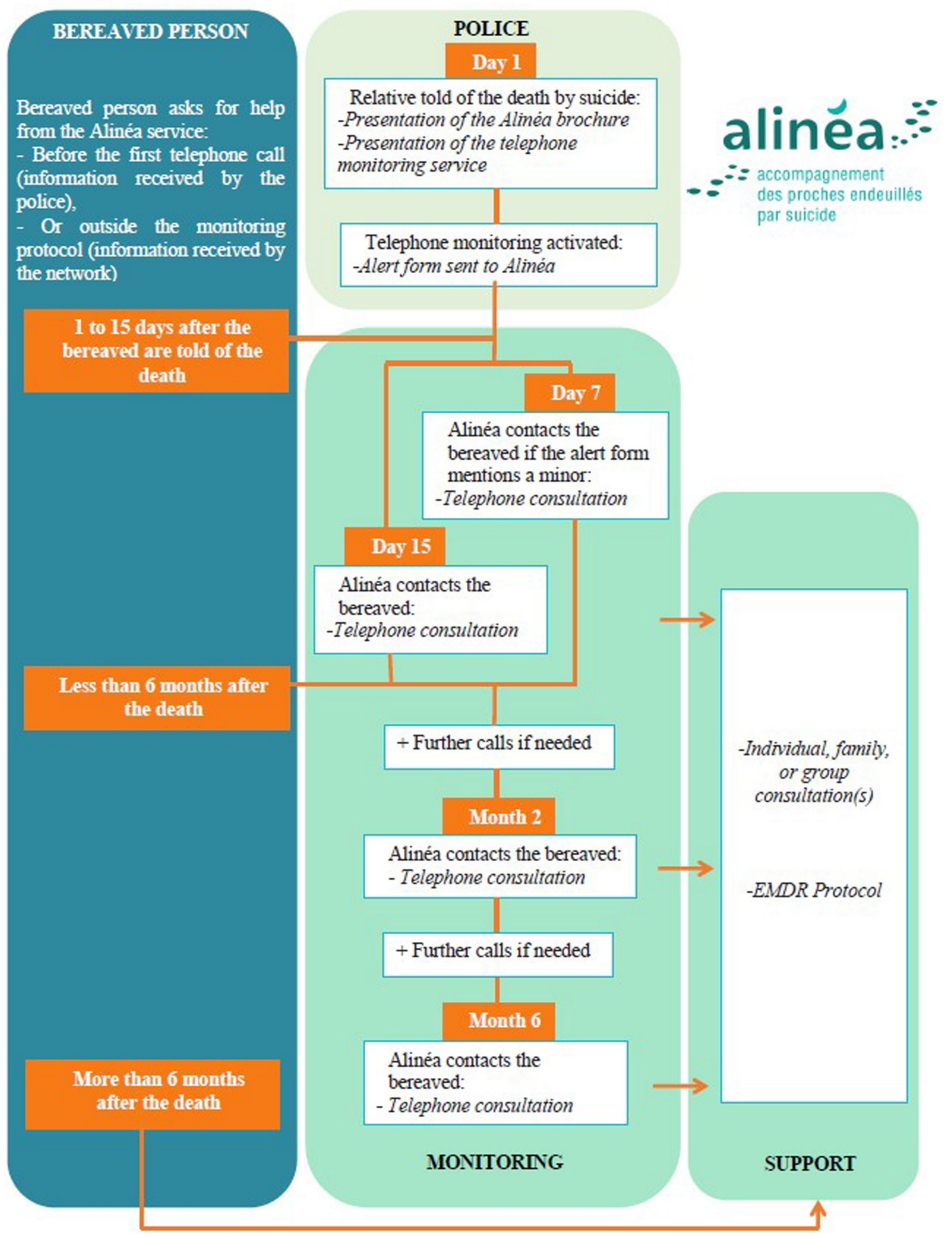
The Alinéa protocol: monitoring and support service for people bereaved by suicide.

The Alinéa protocol was drawn up in the light of research findings and the experience of experts and practitioners in managing potentially traumatic threats to mental health, especially bereavement due to suicide (Cook, 2017; Hobfoll et al., 2021). Figure 2 outlines the Alinéa protocol.

### Initial contact by Alinéa

Alinéa counselors contact the people listed on the alert sheet within 10–15 days of the death, or within 7 days if the list includes children. This initial contact begins with the counselor presenting himself or herself and explaining the reason for the call. Counselors use the ensuing conversation to collect predefined information aimed at establishing a clinical evaluation, understanding family relationships, determining the administrative procedures being followed, and assessing the resources available. Special attention is given to the person who discovered the body and to how the death was announced. If the people contacted are not available to talk at that time, the counselor arranges an appointment for a telephone conversation at a later date.

For example: *P-1 discovered the body of his friend who had committed suicide at home. He called the emergency services and the police. At the end of the investigation process, the police classified the death as suicide to P-1 and the deceased’s two children. At the death announcement, the police informed them of the existence of Alinéa as a professional system that supports relatives bereaved by suicide. P-1 and the children of the deceased agreed to be contacted and supported by Alinéa. Alinéa received the alert form within 48 h of the announcement and began monitoring 10 days after the death.*

### Programmed monitoring calls

Every bereaved person receives two follow-up calls, one 2 months after the death and one 6 months after the death, even if the initial contact was unsuccessful. Hence, the telephone monitoring phase lasts for at least 6 months, at the end of which, the bereaved are told that the telephone monitoring has come to an end but that they can continue receiving calls if they feel they need to. Support continues after 6 months if the bereaved person and counselor agree that it is necessary.

### Flexible monitoring calls

Counselors may offer to call a bereaved person more frequently, depending on his or her clinical condition or wishes. The frequency of these calls is flexible and decided by the bereaved person in conjunction with the counselor. These conversations enable counselors to monitor the bereaved person’s clinical condition.

### Calls from the bereaved

The information leaflet given by police contains a telephone number that relatives can call or text before they receive the initial call from Alinéa and at any time between subsequent monitoring calls. People who lost a loved one to suicide in the past or who are not mentioned on the alert sheet can also ask Alinéa for help (passive postvention). Depending on the results of a clinical evaluation, the Alinéa counselor offers support, either by telephone or face-to-face.

For examples: *P-2 discovered the body of her husband. After a judicial investigation, the police confirmed the circumstances of the death and presented the Alinéa system. P-2 immediately called Alinéa to be guided in announcing the death of her husband to her two children. For 18 months, this wife and mother was supported by Alinéa through telephone consultations. One of the two children was seen in consultation with a psychologist.*

*P-3 lost her son to suicide 6 years ago. She has so far received help from peer associations that introduced her to Alinéa. She calls Alinéa saying that she is “eaten up with guilt.” This grieving mother is supported in individual consultations.*

### Specific support

Alinéa offers face-to-face consultations to suicide survivors presenting complications linked to their grief or who are not comfortable talking by phone. Consultations may be in the form of individual sessions or family sessions. If necessary, the counselor may offer eye movement desensitization and reprocessing therapy targeting the traumatic memory.

### A year after the death

We are currently examining the possibility of extending Alinéa by adding a stage a year after the death. This stage would involve sending the bereaved a letter and a packet of seeds to plant in memory of the deceased and as a reminder that support remains available.

### Group support

The Alinéa project has received a research grant to investigate creating a support group for bereaved persons to complement the support group services already provided by suicide bereavement charities.

### Letter to suicide survivors’ family doctors

Family doctors are key partners in supporting people bereaved by suicide. A step ensuring every bereaved person’s family doctor receives an information letter will be added to the Alinéa protocol.

## Operational strategies

Alinéa had to meet certain conditions before it could be implemented.

### Administrative identity and funding

Alinéa is a psychosocial device piloted by the Fondation Bon Sauveur, a hospital specialized in mental health (Brittany, France). The experimentation and evaluation of Alinéa are funded for 3 years by a Fond d’Innovation Organisationnel en Psychiatrie (French Ministry of Health). At the end of these 3 years, Alinéa is co-funded by the hospital that pilots it and the Agence Régionale de Santé Bretagne (public administrative establishment of the French state responsible for implementing health policy in its region). Thus, the services offered by Alinéa are free of charge for its beneficiaries.

### Legal framework

Alinéa worked with a specialist law firm to draw up a legal framework that complies with all relevant legislation and respects all the parties involved. Thus, every step of the monitoring process, from distributing the information leaflet and collecting the bereaved’s details to providing information and calling the bereaved, is governed by contracts stipulating the partnerships involved and the sharing of data, signed by the region’s attorney general, prefect, police department and gendarmerie, and the Alinéa pilot hospital. These contracts were validated by Agence Régionale de Santé Bretagne.

### Implementation requirements

Before implementing Alinéa we had to codesign the program in conjunction with users, train police how to break the news of a death, and involve the regional health authority in rolling out the protocol.

### Operational framework

Alinéa is run under the supervision of a steering committee comprising the program’s coordinator and partners, who study possible ways of modifying the program in the light of feedback from users. The steering committee makes its decisions in consultation with local health authorities, support and counseling charities, peer counselors who have themselves experienced bereavement by suicide, and funeral directors.

### Program professionals

Program professionals include program coordinators (director and local coordinator), regulators/integrators (role in line with the strategy of existing suicide prevention and suicide bereavement support services), the program’s nurses and psychologists trained in suicidology and psychotraumatology.

*Operational partners* include law enforcement, who receive training during annual visits to police units. Alinéa also supplies specially created tools to help police break the news of a death by suicide and complete the alert process.

### Actions

Alinéa follows what Affeltranger et al. (2018) called the principle of proportionate universalism, which involves adopting a global, rational, and partnership-based approach to “provide universal interventions aimed at all people but with modalities or intensities that vary according to needs.”

## Implications for research

Using a combination of systematized and free data to model the Alinéa program will improve knowledge of grief after suicide. Studies have been carried out to highlight the unique nature of grief after suicide, determine the suicide-bereaved’s specific needs, and measure Alinéa’s effectiveness. This work, which has included clinical field studies, has involved several types of research, notably:

Evaluation research: the general objective of the study is to evaluate the impact of the Alinéa system on users (clinical effectiveness) as well as the processes implemented within the system in order to identify the best operational practices in the proposed intervention approach. The purpose of the study is to restore an intervention model, based on the specificities developed within the Alinéa system, which will serve as a basis for the dissemination and generalization of the system in other territories. This research is currently being finalized for publication.

Basic research: for example, a thesis is in progress, focusing on the experiences of relatives bereaved by suicide, and also the experiences of first responders at death scenes, such as law enforcement and funeral services.

Action research aimed at using feedback from the program’s users to continue the innovation process. For example, one research focused on the co-design of a support group for parents of children bereaved by suicide.

## Conclusion

The sudden, violent, self-inflicted death that is suicide disorganizes the bereaved’s individual, family, and social systems while setting in motion a new system (Andriessen et al., 2017), that of after-suicide, involving legal and funerary procedures. These procedures mark the beginning of the grieving process and the reshaping of the deceased’s family and social environment. They also bring together suicide survivors and first responders within a new system. As a survivor said: “*The policewoman came to tell me that my daughter had died. She stayed with me and told me several times, ‘do not stay alone’, ‘do not stay alone’. Today, that’s all I have left of that horrible moment*.” As a police officer says about his experience: “*you are there, you are them but not them at the same time. They have integrated you because you can be there for several hours and after several hours, well you are no longer a stranger in fact, you are still a police officer but they talk to you about family stuff, all that while you are starting from nothing. It is huge in such a short time in fact. Really a few hours*.”

The co-design approach involves members of the community, namely people who have had the experience, partners and healthcare professionals. The co-created intervention reflects the experiences, lifestyles, beliefs and knowledge of each person (Grattidge et al., 2023). The support of future beneficiaries and collaborators and the implementation of the system are thus facilitated or even enabled (Cluley et al., 2022). As a relative testifies: “*the trauma that I experienced, before talking to [Alinéa’s professional] I had not talked to anyone. (…) If Alinéa had not called me, I would not have gone. (…) Now I can talk, it feels good, of course (…) It is very comforting. (…) Finally, I could be myself*.” Another relative says: “*when I was told that there was someone who specialized in suicides, (…) I said yes because I told myself that it would be specific. (…) I was in a total fog and from the first meeting [Alinéa’s professional] brought some light back into my grey sky. (...) I think [Alinéa’s professional] welcomed it with all the seriousness of things. I came out of it with the impression of being much less overwhelmed*.” As a police officer says: “*the Alinéa system, at the end of the intervention, is a real crutch for me because I do not feel like I’m abandoning them. We do not leave them alone. We stay until the end of the intervention, we try to answer their questions, and [Alinéa] behind, [Alinéa] intervene on top of that (...). It’s a global approach. (…) I think that [Alinéa] help the families as much as [Alinéa] help us. It allows us to work more calmly*.”

Given this new system, beyond its co-designing, what are the essential characteristics of an early proactive collaborative program for people bereaved by suicide? What conditions must the program meet? To identify these characteristics and constraints, it is necessary to model the program as an actor within its surrounding system. Organizational sociology, especially Actor-Network Theory, allows us to “study the content at the same time as the context, while hoping that crisis situations will reveal links that cannot be seen in stable situations” (Akrich et al., 2013). According to the organizational sociology approach, actors’ reactions in a situation lie between two endpoints— “chance” and “necessity” —with an entire spectrum of possibilities between them (Amblard et al., 2004). Amblard et al. (2004) suggest that the ways in which actors respond to a situation depend both on the interdependencies between them, which shape each actor’s stance, and on the degree of uncertainty each actor feels. Faced with an unexpected and potentially traumatic event such as suicide, the notions of interdependence and uncertainty are central both for the victim’s loved ones and for first responders. Perceptions of interdependence and uncertainty govern whether a person feels able to do something, notably establish a protective link with another person. Alinéa is designed to help all those impacted by a suicide to generate this protective link. Indeed, one of the aims of collaborative codesign processes is “to bring together all the bodies who contribute to an issue” to transform a statement that is initially intelligible only to some into a statement that is intelligible to all (Amblard et al., 2004).

## Data Availability

The original contributions presented in the study are included in the article/supplementary material, further inquiries can be directed to the corresponding author.
